# F-Type Lectins: A Highly Diversified Family of Fucose-Binding Proteins with a Unique Sequence Motif and Structural Fold, Involved in Self/Non-Self-Recognition

**DOI:** 10.3389/fimmu.2017.01648

**Published:** 2017-11-29

**Authors:** Gerardo R. Vasta, L. Mario Amzel, Mario A. Bianchet, Matteo Cammarata, Chiguang Feng, Keiko Saito

**Affiliations:** ^1^Department of Microbiology and Immunology, Institute of Marine and Environmental Technology, University of Maryland School of Medicine, University of Maryland, Baltimore, Baltimore, MD, United States; ^2^Department of Biophysics and Biophysical Chemistry, School of Medicine, Johns Hopkins University, Baltimore, MD, United States; ^3^Department of Neurology, School of Medicine, Johns Hopkins University, Baltimore, MD, United States; ^4^Department of Earth and Marine Sciences, University of Palermo, Palermo, Italy; ^5^Department of Marine Biotechnology, Institute of Marine and Environmental Technology, University of Maryland Baltimore County, Baltimore, MD, United States

**Keywords:** F-type lectins, fucolectins, structural modeling, glycan recognition, fucose-binding, self/non-self-recognition, innate immunity

## Abstract

The F-type lectin (FTL) family is one of the most recent to be identified and structurally characterized. Members of the FTL family are characterized by a fucose recognition domain [F-type lectin domain (FTLD)] that displays a novel jellyroll fold (“F-type” fold) and unique carbohydrate- and calcium-binding sequence motifs. This novel lectin family comprises widely distributed proteins exhibiting single, double, or greater multiples of the FTLD, either tandemly arrayed or combined with other structurally and functionally distinct domains, yielding lectin subunits of pleiotropic properties even within a single species. Furthermore, the extraordinary variability of FTL sequences (isoforms) that are expressed in a single individual has revealed genetic mechanisms of diversification in ligand recognition that are unique to FTLs. Functions of FTLs in self/non-self-recognition include innate immunity, fertilization, microbial adhesion, and pathogenesis, among others. In addition, although the F-type fold is distinctive for FTLs, a structure-based search revealed apparently unrelated proteins with minor sequence similarity to FTLs that displayed the FTLD fold. In general, the phylogenetic analysis of FTLD sequences from viruses to mammals reveals clades that are consistent with the currently accepted taxonomy of extant species. However, the surprisingly discontinuous distribution of FTLDs within each taxonomic category suggests not only an extensive structural/functional diversification of the FTLs along evolutionary lineages but also that this intriguing lectin family has been subject to frequent gene duplication, secondary loss, lateral transfer, and functional co-option.

## INTRODUCTION

Recognition of glycans exposed on the surface of microbial pathogens and parasites by the host’s cell-associated and soluble lectins is considered the initial key step in the innate immune response of both invertebrates and vertebrates (1–5). Members of several lectin families characterized by unique sequence motifs and structural folds such as C-type lectins (CTLs) (6), peptidoglycan binding proteins (7), ficolins (8), pentraxins (PXNs) (9), galectins (10), and most recently, F-type lectins (FTLs) (11–14) have been implicated in immune surveillance and homeostasis. However, the participation of these and other lectin families in multiple intra- and extracellular functions including folding, sorting, and secretion of glycoproteins, cell–cell interactions, and signaling and transport in early development, tissue repair, and general cell functions, as well as host colonization by microbial pathogens and parasites have also been firmly established (5).

F-type lectins are fucose-binding proteins of wide taxonomic distribution from viruses to vertebrates and constitute the most recently identified lectin family (11–14). They are characterized by a fucose recognition domain [F-type lectin domain (FTLD)] that displays a novel fold (the “F-type” fold) consisting of a β-barrel with jellyroll topology and unique fucose- and calcium-binding sequence motifs (13). Although FTLs can display a single FTLD, sometimes associated with one or more structurally and functionally distinct domains in a single polypeptide, the presence of a variable number of tandemly arrayed FTLDs is also a common occurrence in members of this lectin family. Some FTLs mediate immune recognition (13–16), whereas others are involved in microbial pathogenesis (17–23), fertilization (24–26), and other diverse functions.

The identification of the FTL family was a fortuitous discovery that resulted from the search for fucose-binding CTLs in serum and liver extracts from the striped bass (*Morone saxatilis*) (11, 12). Affinity chromatography on l-fucose-Sepharose yielded a 32 kDa protein (MsaFBP32) that did not require calcium or other divalent cations for binding to cells. Partial Edman sequencing of the protein enabled cDNA and genomic cloning and revealed the presence of two 140-amino acid tandemly arrayed domains. Analysis of the deduced polypeptide sequence of MsaFBP32 failed to identify the signature motif of the CTLs or any of the known lectin families described at the time and suggested that MsaFBP32 represented a novel lectin type. Although no matches to known lectins were initially identified, the search of sequence databases revealed a stretch of N-terminus sequence from a single protein named PXN1-XENLA (27) that shared significant similarity to the MsaFBP32 lectin motif. Surprisingly, PXN1-XENLA, which is described as a PXN-fusion protein cloned from the liver of the African clawed frog (*Xenopus laevis*), consists of an MsaFBP32-like domain linked to a PXN domain that also exhibits lectin activity (28). Furthermore, a search of *X. laevis* and *Xenopus tropicalis* EST databases revealed additional FBPLs different from PXN1-XENLA, with multiple FBPLs. The information obtained enabled the cloning of similar lectins in several fish species and later the *in silico* identification of FBPLs in the growing number of EST and genomic databases for multiple invertebrate and vertebrate species, mostly fish and amphibians (11, 12). Surprisingly, three FBPL tandemly arrayed sequences were identified in the SP2159 ORF from the genome of the capsulated and virulent strain (TIGR4) of *Streptococcus pneumoniae* (11, 12). As a whole, this experimental and *in silico* effort led to the identification of the novel lectin family (FTL family) characterized by proteins present in both prokaryotes and eukaryotes, which displayed the newly identified lectin domain (FTLD), either tandemly arrayed or in mosaic combinations with other structurally and functionally distinct domains (11, 12).

Structural studies were initiated with the simplest FTL family member carrying a single FTLD, the European eel agglutinin [*Anguilla anguilla* agglutinin (AAA)] (13). These were followed by the FTL from the striped bass (*M. saxatilis*; MsaFBP32) that carries two tandemly arrayed FTLDs (14). The resolution of structures for the AAA and MsaFBL32 complexed with fucose enabled the identification of a novel structural fold (the F-type fold) and the amino acid residues in the carbohydrate recognition domain (CRD) that interact with the fucose ligand, as well as with the subterminal sugar units in fucose-containing oligosaccharides. Furthermore, a fold-based search [Dali database (29)] revealed several proteins display the F-type fold, but that only share negligible sequence homology with FTLs, including discoidins, clotting factors, and fungal and bacterial glycoenzymes (13). This information enabled not only the formulation of proposals about the possible evolutionary origin of the FTLD but also about its functional co-option along vertebrate lineages.

In later functional studies, the biological characterization of FTLs from teleost fish revealed their capacity for pathogen recognition and their roles as opsonins in innate immunity, the characterization of the gene products from the identified *Streptococcus* spp. FTL sequences as virulence factors (lectinolysins) (20–22), and the identification of sperm acrosomal proteins (bindins) from the oyster *Crassostrea gigas* as extremely diversified FTLs with role(s) in fertilization (24–26). In recent years, the exponentially growing number of sequenced genomes from multiple species, ranging from viruses to pro- and eukaryotes has enabled the identification of FTLs in additional taxa, thereby greatly expanding our knowledge about the distribution of the FTLD in nature. In this regard, a rigorous and exhaustive computational study has recently provided significant insight into the taxonomic prevalence of the FTLD (30). Finally, functional studies aimed at elucidating the role(s) of FTLs in innate immunity using the invaluable resources available for the genetically tractable zebrafish model system are ongoing. In the following sections, the most relevant structural and functional aspects of the FTL family are discussed.

## STRUCTURAL ASPECTS

**Figure 1:**
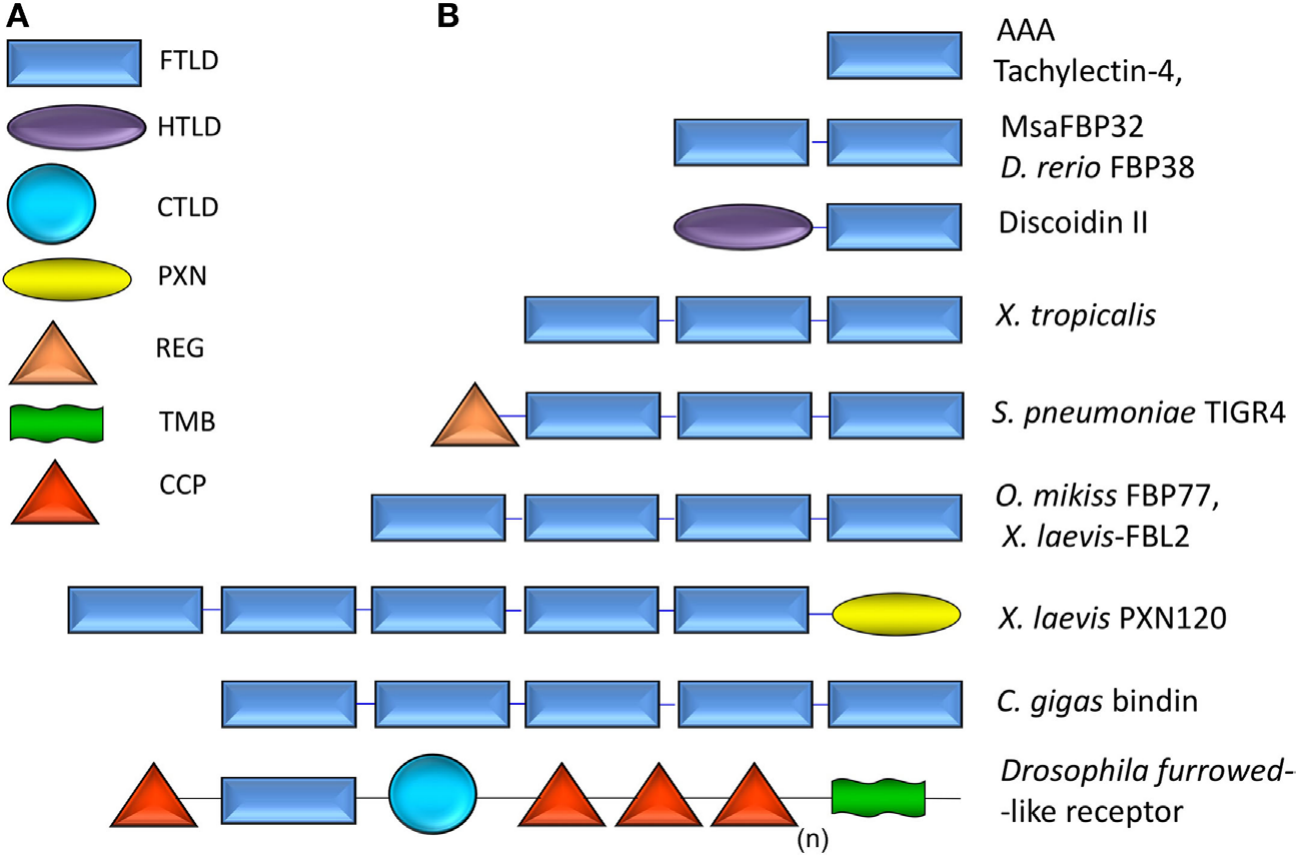
Domain organization of F-type lectins (FTLs) from prokaryotes, invertebrates, and vertebrates: **(A)** schematic illustration of selected domain types found in FTLs: FTLD, F-type lectin domain; HTLD, H-type lectin domain; CTLD, C-type lectin domain; PXN, pentraxin; REG, regulatory; TMB, transmembrane domain; and CCP, complement control protein domain. **(B)** Schematic illustration of the domain organization in selected examples of FTLs (1–5 FTLDs and chimeric molecular species) described in prokaryote, invertebrate, and vertebrate species. The subscript “n” indicates the extended number of CCP domains present in furrowed [adapted from Ref. (11, 12)].

The sequence alignment of the *M. saxatilis* FTL (MsaFBP32) and *X. laevis* PXN-fusion protein (PXN1-XENLA) led to the identification of an approximately 140-amino acid long lectin domain and a tentative amino acid sequence motif common to a number of lectins, as well as selected domains present in sequences that had been described in other contexts such as the *Drosophila* furrowed gene and the *Streptococcus* fucose regulon. In turn, this resulted in the identification of a novel fucose-binding lectin family (FTL family) that included both prokaryotes (*S. pneumoniae TIGR4*) and eukaryotes (*Drosophila*, fish, amphibians, and others) (11, 12) (Figure 1). The resolution of the structure of the AAA–fucose complex revealed a new lectin fold (FTL fold) and identified the amino acid residues that interact with the non-reducing terminal fucose and coordinate the divalent cation and model those that are established with the subterminal sugar units of an oligosaccharide ligand (13). In turn, this structural information led to the rigorous identification of the FTL fucose- and calcium-binding sequence motifs (13).

### FTL Fold

**Figure 2:**
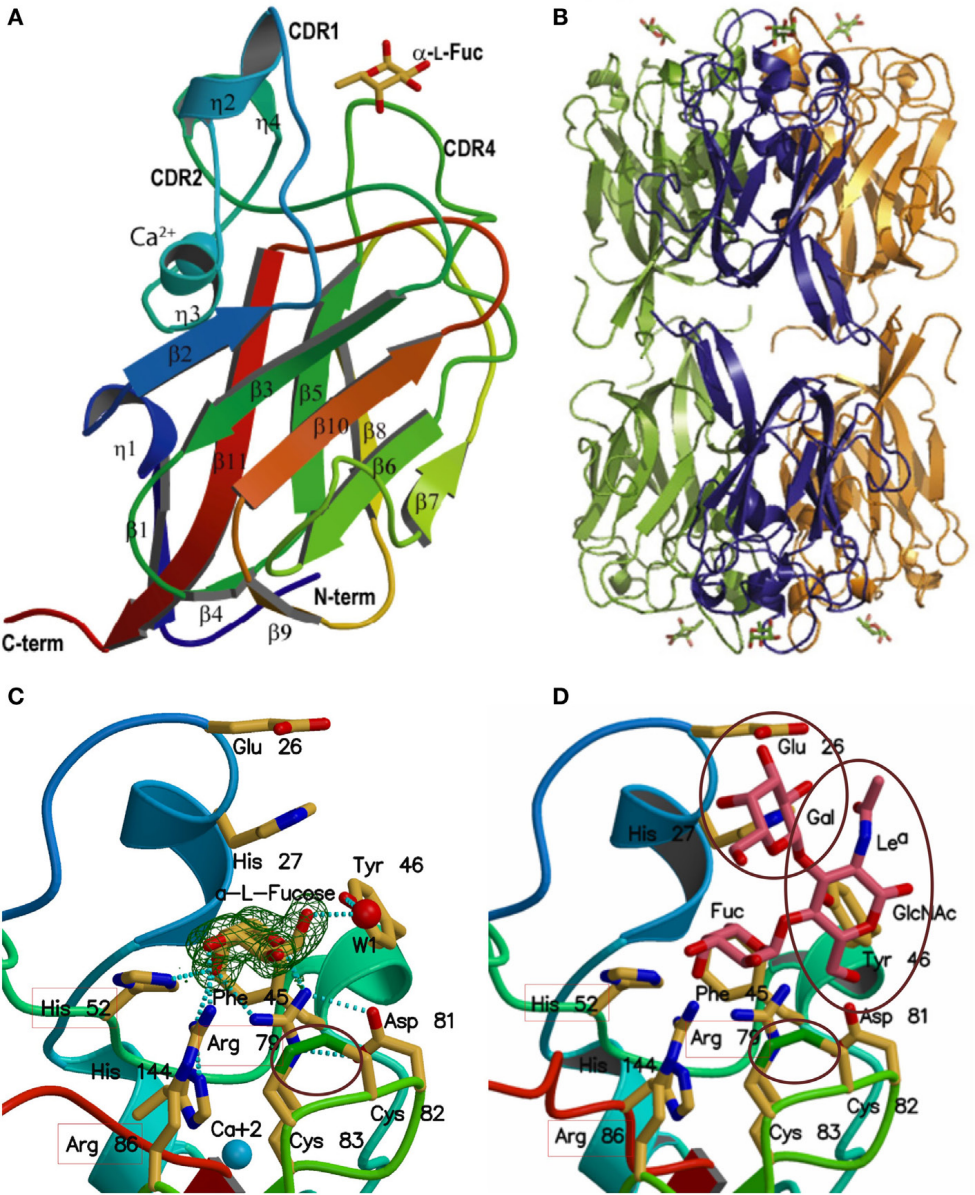
Structure of *Anguilla anguilla* agglutinin (AAA) and quaternary structure of AAA oligomers. **(A)** Ribbon diagram of AAA showing the two β-sheets, the loops (CDRs) encircling the binding site, and 3_10_ helices. Bound α-l-fucose is shown as a stick model above the lectin in yellow. Calcium is shown as a blue sphere. **(B)** Quaternary structure of the AAA hexamer: in each trimer a single chlorine ion coordinated by Lys^16^ from each subunit determines the three-fold axis of rotation, and the hexamer is formed by two stacked trimers with opposing carbohydrate-binding faces. **(C)** Primary binding site: interactions of the AAA binding site with l-fucose: the three basic amino acid residues that interact with the axial OH on C4 are indicated with the red boxes. The interaction of the disulfide bond (Cys^82^-Cys^83^) with the C1–C2 bond of the l-fucose is indicated with a circle. **(D)** Extended binding site: model of the interactions between AAA and a terminally fucosylated Le^a^ trisaccharide. Interactions on the protein with the l-Fuc are indicated as in **(C)** above. Subterminal GlcNAc and Gal are indicated by purple circles, and the interacting amino acid residues are labeled. See text for details [adapted from Ref. (13, 14)].

The FTL fold, initially described in the AAA/α-l-fucose (α-Fuc) complex (Figure 2), consists of a β-barrel with jelly roll topology comprising two β-sheets of three (β5, β8, and β11) and five (β2, β3, β10, β6, and β7) antiparallel β-strands, respectively, placed against each other (Figure 2A). Two short antiparallel strands (β4 and β9) close the “bottom” of the barrel, from which the N- and C-termini protrude to form an antiparallel two-strand β-sheet (13). On the “top” face of the barrel, the connecting β-strands from the opposite sheets form five loops (CDR1–5) that surround the heavily positively charged pocket that binds the α-Fuc (Figure 2A). CDR1 is the most protruding loop, and at its exposed apex Glu^26^ is placed over the aromatic ring of His^27^, and both over the central hollow. At the side of the barrel, a substructure containing three 3_10_ helices (h2, h3, and h4) tightly coordinates a cation (tentatively identified as calcium) *via* seven oxygen atoms of six residues [Asn^35^ (O), Asp^38^ (Od1), Asn^40^ (O), Ser^49^ (O, Og1), Cys^146^ (O), and Glu^147^ (Oe1)] both from the peptide backbone and side chains in a pentagonal bipyramidal geometry. The distance between the cation binding site and the sugar binding pocket indicates that the divalent cation does not directly interact with the carbohydrate as in CTLs, but that together with two disulfide bridges (Cys^50^-Cys^146^ and Cys^108^-Cys^124^) and two salt bridges (Arg^41^-Glu^149^ and Asp^64^-Arg^131^) that clamp the structure together, rather stabilizes the fold and shape the key CDR1 and CDR2 loops (13). The AAA subunits can form chloride-induced trimers that contain one cation (Ca^2+^) per domain and several Cl^−^ placed on the three-fold axis, and two trimers can form hexamers with opposing carbohydrate-binding surfaces (Figure 2B).

### Primary Fucose-Binding Site

The AAA/α-Fuc structure revealed that the protein binds to α-Fuc through hydrogen bonds established between the side chains of three basic amino acid residues (Nε of His^52^ and the guanidinium groups of Arg^79^ and Arg^86^) situated in a shallow cleft and the axial 4-OH of the sugar. Interactions are also established between this basic triad and the ring O5 and equatorial 3-OH of the sugar (13) (Figure 2C). A unique disulfide bridge formed by contiguous cysteines (Cys^82^ and Cys^83^) establishes a van der Waals contact with the bond between C1 and C2 of the α-Fuc ring, and the C6, which fits into a hydrophobic pocket formed by His^27^ and Phe^45^, together with Leu^23^ and Tyr^46^ (13). As AAA can also recognize 3-*O*-methyl-d-galactose and 3-*O*-methyl-d-fucose, sugars that display similar key configurational features of α-Fuc (i.e., axial hydroxyl and hydrophobic moiety), it becomes clear that as for most animal lectins, the specificity of AAA for α-Fuc is nominal rather than absolute.

### Extended Carbohydrate-Binding Site

**Figure 3:**
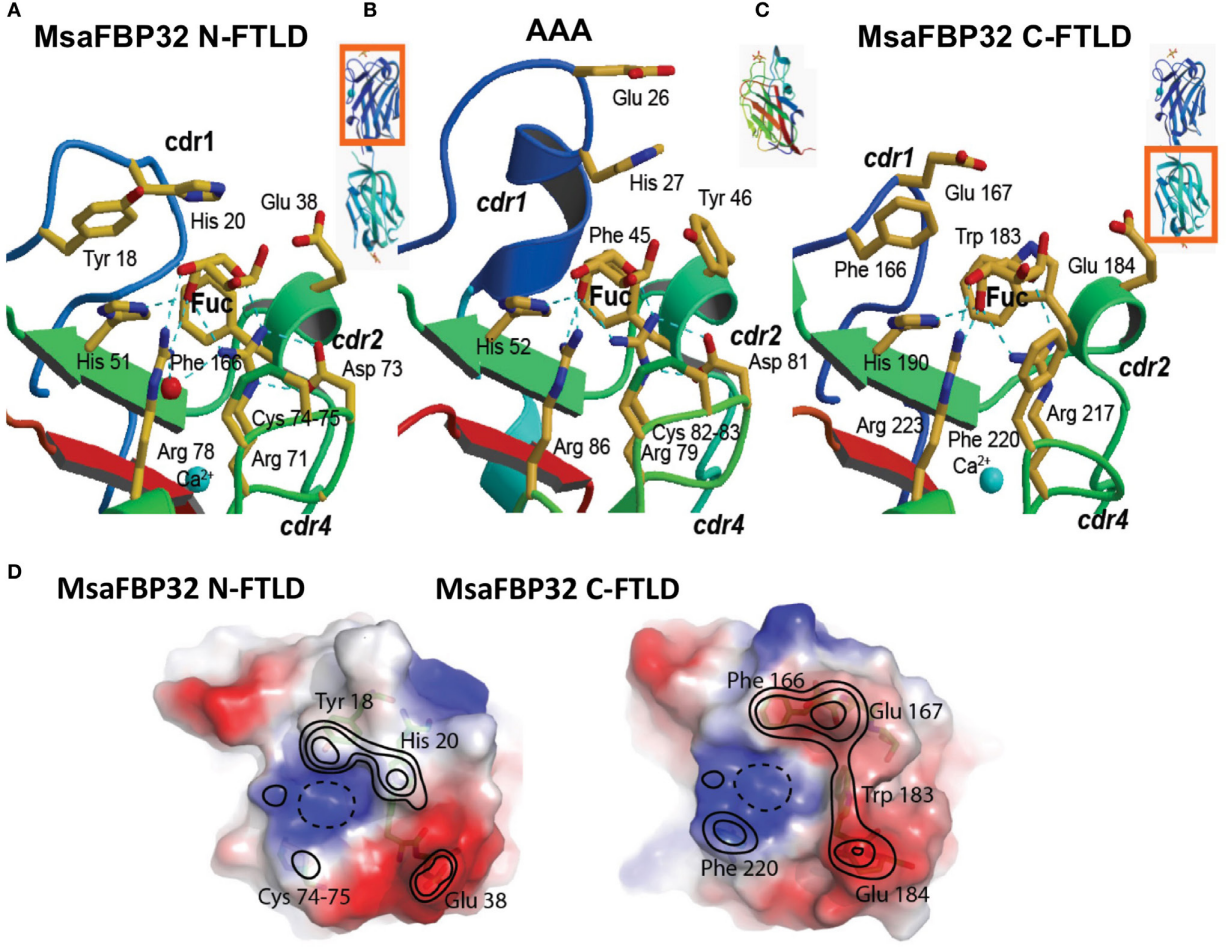
Binding sites of the binary F-type lectin (FTL) MsaFBP32. **(A–C)** Comparison of binding sites with *Anguilla anguilla* agglutinin (AAA): side-by-side comparison of fucose recognition by the striped bass N-terminal FTL domain (N-FTLD) **(A)** and C-FTLD **(C)** with the AAA FTLD **(B)**. See the text for description of differences and similarities. **(D)** Topology of the N-FTLD and C-FTLD carbohydrate recognition sites in MsaFBP32. The surfaces were colored based on their electrostatic potential at the surface. Contour levels represent approximated elevations from the bottom of the binding site (drawn with broken lines), where the axial 4-OH of the fucose binds [adapted from Ref. (13, 14)].

The AAA/α-Fuc structure also enabled the modeling of potential interactions between the protein and α-Fuc-containing oligosaccharides such as H and Lewis moieties that are specifically recognized through interactions with amino acid residues located in the so-called “extended binding site” (13) (Figure 2D). AAA recognizes blood group H type 1 (Fucα1-2 Galβ1-3GlcNAcβ1-3Galβ1-4Glc) and Le^a^ (Galβ1-3[Fucα1-4]GlcNAcβ1-3Galβ1-4Glc) oligosaccharides *via* additional interactions established between amino acid residues in CDRs 1–5 that encircle the binding cleft, with the subterminal units of the H1 and Le^a^ trisaccharides. Specifically, Glu^26^ and His^27^ on CDR1 can interact with hydroxyls (3-OH and 2-OH) in Gal and oxygen of the GlcNAc 2-N-Acetyl group in Le^a^, or GlcNAc 6-OH and 4-OH groups in H. The OH group of Tyr^46^ in CDR2 can interact with the glycosidic bond oxygen between Gal and GlcNAc moieties. Furthermore, Asp^81^ and Arg^79^ in CDR4 interact with the GlcNAc 6-OH group in Le^a^, and a water molecule can bridge the Gal 4-OH group with Asp^81^ in H1. The rigidity of the CDR1 loop prevents recognition of Le*^x^*, in which the 2-*N*-acetyl is pointed toward the Fuc side of the oligosaccharide (13). In contrast, MsaFBP32, an FTL that displays a shorter CDR1 loop (Figure 3A), would have a broader specificity for Le oligosaccharides (14).

### Carbohydrate and Cation Binding Sequence Motifs

From both the initial sequence alignment of MsaFBP32 (11, 12) and analysis of the binding site structure of AAA (13) described above, highly conserved sequence motifs for carbohydrate and calcium binding were identified in most FTL sequences available at that time. The fucose-binding sequence motif was defined as: His followed 24 residues downstream by a segment of sequence that starts with an arginine followed one residue apart by a negatively charged residue, which salt-bridges the preceding arginine and ends with a basic residue [HX_24_RXDX_4_ (R or K), where X indicates any amino acid residue]. Loops participating in the hydrophobic pocket for the fucose methyl group, such as CDR2, are also conserved in both hydropathic profile and length. The cation binding sequence motif is h_2_DGx, where h indicates a small hydrophobic amino acid residue (i.e., V, A, or I) and x stands for a small hydrophilic residue (i.e., N, D, or S). Three of the seven oxygens that bind the cation are contributed by this motif, which in AAA is located just after the 3_10_-helix h3 (13).

Some FTLDs, however, deviate from the fucose-binding sequence motif, and the changes may suggest either different specificities or loss of sugar-recognition activity. In *Drosophila* CG9095, two amino acid residues of the basic triad are replaced by aliphatic residues, which are unlikely to establish the hydrogen bonds typical of the canonical FTLD (11, 12). Furthermore, most duplicate tandem FTLs such as MsaFBP32 possess a unique combination of sugar-binding motif in which although the triad of basic residues that interact with the sugar’s axial hydroxyl on C4 is conserved in both FTLDs, one domain has lost the disulfide bond from the contiguous cysteines (Cys^82^ and Cys^83^ in AAA). Similarly, replacements of metal-coordinating residues are frequent, and those that occur at Ser^49^ are of special interest, since as a bidentate ligand, it is central to the coordination geometry. In most cases, this position is substituted by residues that are able to form similar coordination bonds, such as Asp, Gln, Glu, Thr, and Tyr, but in some sequences this is not the case. In the latter, a water molecule may substitute in cation coordination, or it is possible that the coordination geometry is modified (11, 12).

In general, sequence insertions or deletions (indels) are permissible as long as any potentially disruptive effect on the core fold is minimal, as in the FTLD CDRs, where most indels are present. Interestingly, the CDR1 loop, which interacts with subterminal sugar units, shows considerable divergence suggesting that it might determine the fine specificity for a wide diversity of glycoconjugates. Coincidentally, in the MsaFBP32 gene, the exons coding the two FTLDs are split by introns localized at the lower side of the barrel close to a turn that is also variable in length (in AAA: Glu^123^-Cys^124^) and would not be subject to junctional diversity during splicing (11, 12).

### Tandemly Arrayed FTLDs Are Similar but Not Identical

Alignment of the amino acid sequences of the N-terminal FTLD (N-FTLD) and C-terminal FTLD (C-FTLD) of MsaFBP32 revealed that they are similar but not identical (11, 12). Sequence of N-FTLD is closer to AAA than the C-FTLD, suggesting that they display different carbohydrate specificity (11, 12). The structure of the MsaFBP32/l-Fuc complex revealed that the overall structure of the N-FTLD is similar to that of the C-FTLD and that recognition of l-Fuc by each FTLD is mediated by a repertoire of polar and apolar interactions similar to those observed in AAA (Figure 3). However, in both N- and C-FTLDs of MsaFBP32, the pocket for the C6 is more solvent accessible than that in AAA due to the shorter CRD1 (14). In addition, significant differences were observed between the binding sites of the MsaFBP32 N- and C-FTLDs. The C6 pocket in the C-FTLD binding site is less open than in the N-FTLD binding site due to the replacement of Phe^37^ by the bulkier Trp^183^ and the replacement of apolar contact of the S–S bridge with l-Fuc observed in AAA and N-FTLD by a bulkier Phe^220^ that partially displaces the sugar from the shallow binding pocket (14) (Figures 3A–C). Furthermore, an examination of the topology and surface potential of the primary and extended binding sites reveals significant differences in the N- and C-FTLDs, specifically in the extended binding site (14) (Figure 3D), suggesting that the N-FTLD binding site recognizes more complex fucosylated oligosaccharides, with a relatively higher avidity than the C-FTLD. For example, in the N-FTLD, a methyl group of a second fucose may dock on top of Phe^37^, but in the C-FTLD, Trp^183^ closes the pocket with its indole ring, thereby interfering with the second fucose unit in Lewis tetrasaccharides (14).

### FTL Isoforms and Diversity in Ligand Recognition

The presence in single individuals of multiple FTL isolectins, which display sequence replacements at positions that are critical for sugar recognition, strongly suggests diversity in carbohydrate specificity, a feature that is key not only for proteins involved in innate immunity, such as in the eel FTLs (15), but also for those that recognize heterogeneous “self” glycan ligands, as proposed for the Pacific oyster *C. gigas* bindins (24–26). It should also be kept in mind, however, that our knowledge about regulation of expression of FTLs in both immune and developmental processes is very limited at this time.

#### FTL Isoforms in AAA and the Japanese Eel

**Figure 4:**
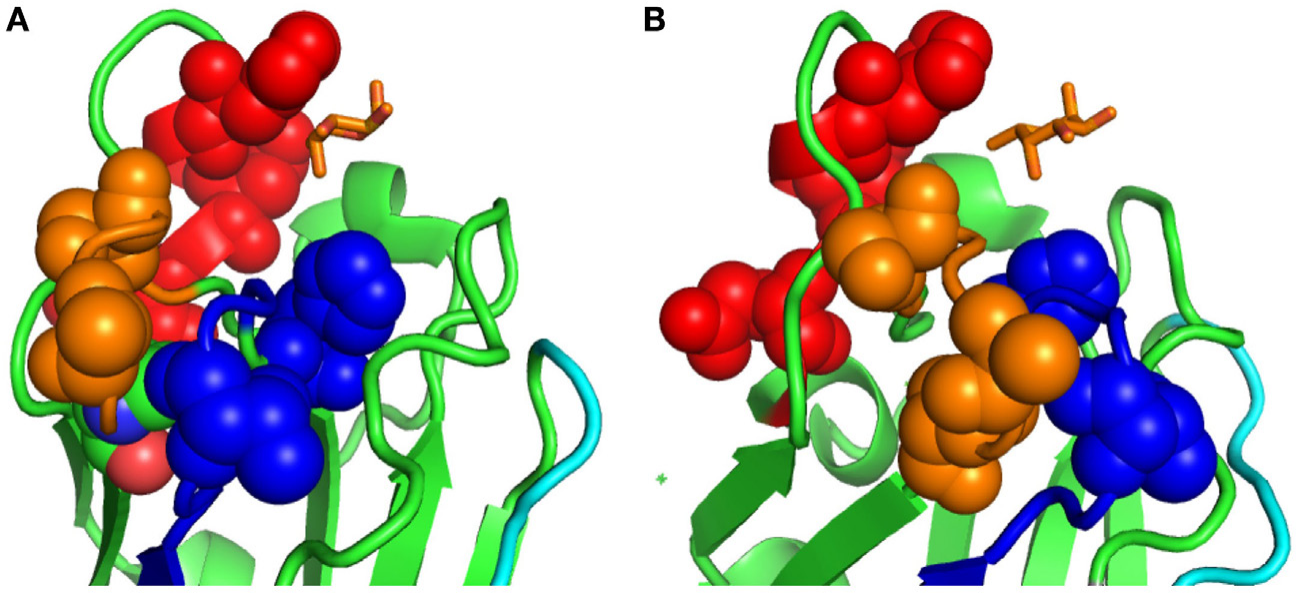
Sequence variability in the loops surrounding the binding cleft of the *Anguilla japonica* F-type lectin (FTL) isoforms: **(A,B)** two side views of the residues that show high variability among the eFL-1-7 FTL isoforms in the Japanese eel *A. japonica*. The residues shown correspond to those present at those positions in the *Anguilla anguilla* agglutinin (AAA) structure. Sequence alignment of the AAA with eFL-1-7 isoforms shows high variability in the loops CDR1 and CDR2, with the amino acid replacements at several positions suggesting a broader specificity for some FTL isoforms.

Although the structural analysis of the predominant sequence in the AAA and MsaFBP32 crystals revealed that the number of carbohydrate moieties specifically recognized by these lectins is limited, the expression of multiple isoforms with amino acid substitutions at key positions for sugar binding significantly broadens the range of recognized ligands (13–15). For example, variability in key sequence positions in the binding cleft and the surrounding loops in the multiple FTL isoforms expressed in the Japanese eel (*Anguilla japonica*) (Figure 4) may expand the range of glycan ligands recognized by the lectin isoform repertoire by the establishment of alternative interactions with terminal and subterminal sugar units of the oligosaccharides (15). The AAA sequence predominant in the crystal shows sequence identities with the seven FTLs from *A. japonica* ranging from 68% to 78%. All FTL sequences from both *A. anguilla* (AAA) and *A. japonica* conserve the basic amino acid triad that interacts with the C4 hydroxyl in fucose, showing strict conservation of His^52^ and the CDR4 sequence (13, 15). CDR1 and CDR2 conserve their size, although they present interesting sequence variations in residues associated with the hydrophobic pocket for the fucose 5-Me and oligosaccharide binding, with CDR1 showing the greatest variability. Most of the isoforms, however, conserve polar residues at the CDR1 apex, probably for interaction with the third moiety of putative oligosaccharide ligands and the two aromatic CDR2 residues in the N-terminus of h4 [like Phe^45^ and Tyr^46^ in AAA (13)] that form the 5-Me pocket. In the isoforms eFL-1 and eFL-5, however, the CDR1 is thinner and more flexible due to smaller residues in the apex of CDR1, thereby the 5-Me hydrophobic pocket more solvent accessible. In eFL-5, sequence replacement by smaller residues in this pocket is maximized, perhaps leading to broader specificity. Furthermore, Ser substitutions of Leu^23^ and Phe^45^ may result in recognition of galactose-containing oligosaccharides, by providing additional polar interactions with the 6-OH (13).

#### FTL Isoforms in the Pacific Oyster

**Figure 5:**
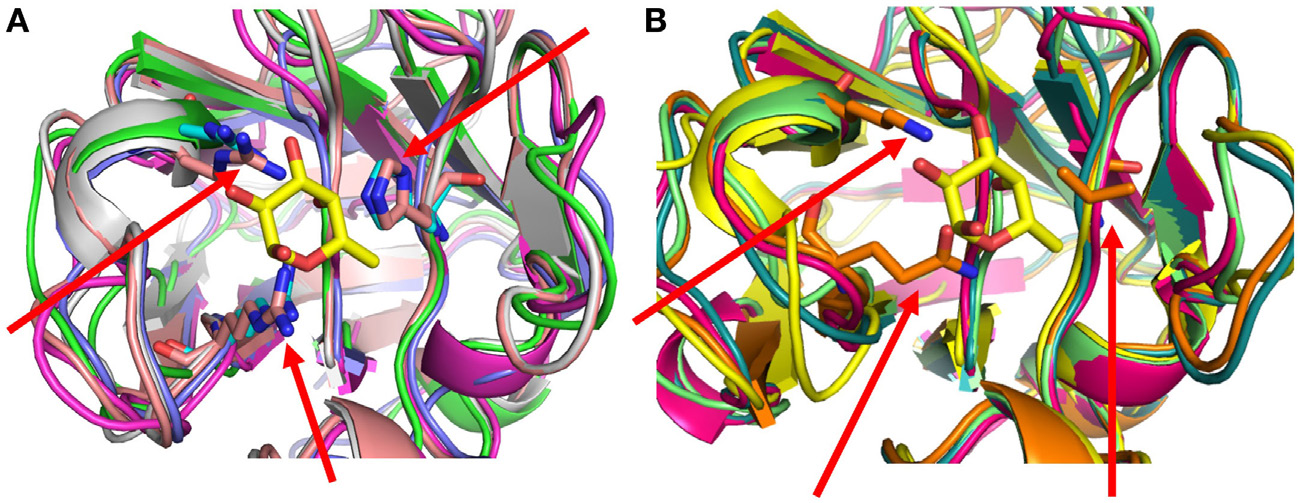
Sequence variability in the loops surrounding the binding cleft of the Pacific oyster *Crassostrea gigas* F-type lectin (FTL) isoforms: **(A)** five *C. gigas* FTL isoforms (#1, 2, 4, 5, and 6) conserve the triad of basic residues (His-Arg-Arg) that in the *Anguilla anguilla* agglutinin structure (His^52^, Arg^79^, and Arg^86^) were shown to interact with the hydroxyl on C4 of L-fuc. Bound α-l-fucose is shown as a stick model in yellow, at the center of the recognition cleft. All five sequences are modeled but for clarity, only the His, Arg, and Arg side chains of two of the models are shown (red arrows). **(B)** Five *C. gigas* FTL isoforms (#3, 7, 8, 9, and 10) lack the triad of basic residues, and these are replaced by different combinations of residues. All five models are shown but only the side chains of the residues of *C. gigas* isoform 7 (Val 39, Gln 66, and Lys 69) are shown (red arrows).

In FTLs (“bindins”) of the Pacific oyster *C. gigas*, the genetic mechanisms for generating diversity in ligand recognition by lectin isoforms have been characterized in detail (24–26). Oyster bindins are gamete recognition proteins present in sperm acrosomes that bond sperm to the egg vitelline envelope during fertilization. Oyster bindins can display from one to five tandemly arrayed FTLDs. Although oyster bindins are encoded by a small number of distinct single copy genes, it appears that oysters have evolved multiple genetic mechanisms to enhance FTLD variability in sperm bindin (24). First, the FTLD repeats have diversified by positive selection at eight sites clustered on the FTL’s fucose- binding pocket, similarly to the *A. japonica* isoforms (Figures 5A,B). It is noteworthy that some *C. gigas* FTL isoforms conserve the triad of basic residues (His-Arg-Arg) that in the AAA structure interact with the hydroxyl on C4 of L-Fuc (Figure 5A), while in other isoforms these residues by other combinations that would be unable to bind fucose at the recognition site (Figure 5B). Second, increased diversity is generated by recombination in an intron that is highly variable in size and sequence located in the middle of each FTLD, to yield many different lectin domain sequences. Finally, alternative splicing in bindin cDNAs can determine the number of repeats (between one and five) per bindin mRNA (24). Interestingly, a retroposon with high homology to reverse transcriptase was identified in a three FTLD gene immediately upstream of the first FTLD repeat, suggesting that retroposition is one mechanism by which F-lectin repeats are duplicated (25, 26). In addition, the identification of a GA microsatellite in each intron, immediately upstream of the start of each FTLD exon and a downstream CT microsatellite, suggests that loopout strand hybridization can occur and that lectin repeats may replicate and transpose within the gene. It is noteworthy that neither the retrotransposon nor the CT microsatellite is present in the single FTLD containing gene (25, 26). In summary, positive selection, alternative splicing, and recombination can generate the most extraordinary intraspecific polymorphism for any known lectin, with potentially thousands of bindin variants with different numbers of FTLDs and distinct carbohydrate specificity. However, male oysters only translate one or two isoforms into protein, yielding sperm cells with potentially bindin preference for selected egg’s vitelline envelopes (24–26).

### Domain Organization of FTLs

The identification of a large number of proteins exhibiting the FTL sequence motif as multiple tandemly arrayed FTLDs enabled the establishment of the FTLs as a novel lectin family (11, 12) (Figure 1). In this regard, the variety of sequences identified as encoding for multiple FTLDs also illustrates the predominance of domain duplication and domain shuffling within the FTL family. Furthermore, identification in both prokaryotes and eukaryotes of mosaic FTLs displaying the FTLD in various combinations with other structurally and functionally domains suggests its extensive functional diversification in the evolution of the FTL family (11, 12). In general, taxonomically consistent domain organization of FTLs can be observed among closely related organisms, although multiple exceptions of unusual domain associations occur, which illustrate the evolutionary and ecological adaptability of this lectin family and potentially frequent lateral transfer along viral, prokaryotic, and eukaryotic lineages (11, 12, 30). It is noteworthy that while prokaryotic FTLs usually display single FTLDs in combination with diverse domains, in eukaryotes the FTLDs occur more frequently in multiple repeats, sometimes also in tandem with other domains (11, 12, 30). Among these distinct domains, carbohydrate-binding domains from other lectin families (CTLs and PXNs), complement control modules (CCP), transmembrane domains, and FA58C domains are the frequently co-occurring domains present in eukaryotic FTLs (11, 12, 30).

From the FTL sequences examined, those from *Drosophila* (CG9095 and *furrowed*) (31), sea urchin (SpCRL) (32), *S. pneumoniae* TIGR4 (11, 12), *Streptococcus mitis* (20, 21), and the amphioxus *Branchiostoma floridae* (30) represent interesting examples of polypeptides that display diverse domains in combination with FTLDs. In *Drosophila*, these domains include complement control domains (CCP), a CTLD, and a predicted transmembrane domain (12, 31). It is noteworthy that in CG9095 the CTLD is unlikely to bind carbohydrate because the canonical residues of the CRD are missing (11, 12) (Figure 1). For the sea urchin SpCRL, domains associated with the FTLD include CCP, S/T/P domain, and factor I-membrane attack complex domain (11, 12). In the *X. laevis* Xla-PXN-FBPL, another mosaic protein, a PXN domain is joined to multiple FTLDs (11, 12, 27, 28). Most interestingly, a hypothetical protein of *Microbulbifer degradans*, a microorganism capable of degrading diverse polysaccharides, has an FTLD that adjoins the structurally analogous F5/8 discoidin domain [FA58C (33)] of coagulation factors. The association of these two analogous domains is intriguing from an evolutionary perspective because they share the same fold (13) despite showing weak sequence homology. It is possible that these domains perform roles analogous to the so-called carbohydrate-binding modules present in microorganisms (34) for which similarities have already emerged (35). The considerable diversity evident from these topologies, in which the binding site motif is strictly conserved, suggests a diverse spectrum of functions fulfilled by specific recognition of l-Fuc in various environments (11, 12, 30).

#### Oligomeric Organization of FTL Polypeptides

Oligomerization of lectin subunits results in multivalency, a property that enables ligand cross-linking and cell agglutination and confers higher lectin avidity for clustered glycans (36). For those lectins such as FTLs that carry multiple CRDs in each polypeptide, these properties are further enhanced by the association of lectin subunits into oligomeric species (13, 14). The physiological structures of AAA are homotrimers and hexamers, which enable cooperative binding to multivalent glycans (13). Like the MBL, the three-fold cyclic symmetry of the AAA trimer would optimize the orientation and spacing of the individual FTLD binding sites for optimal binding to glycan ligand presentation on microbial surfaces. Thus, even if the AAA and MBL recognize the same monosaccharide (in addition to mannose, MBL also binds fucose), the microbial surface glycan architecture recognized by the AAA and MBL trimers is different, as the distances between CRDs in AAA (26 Å) is almost half of that in MBL (45 Å) (13). Therefore, by recognizing different microorganisms, FTLs and CTLs would considerably expand the lectin-mediated recognition capacity in species that are endowed with both lectin types.

**Figure 6:**
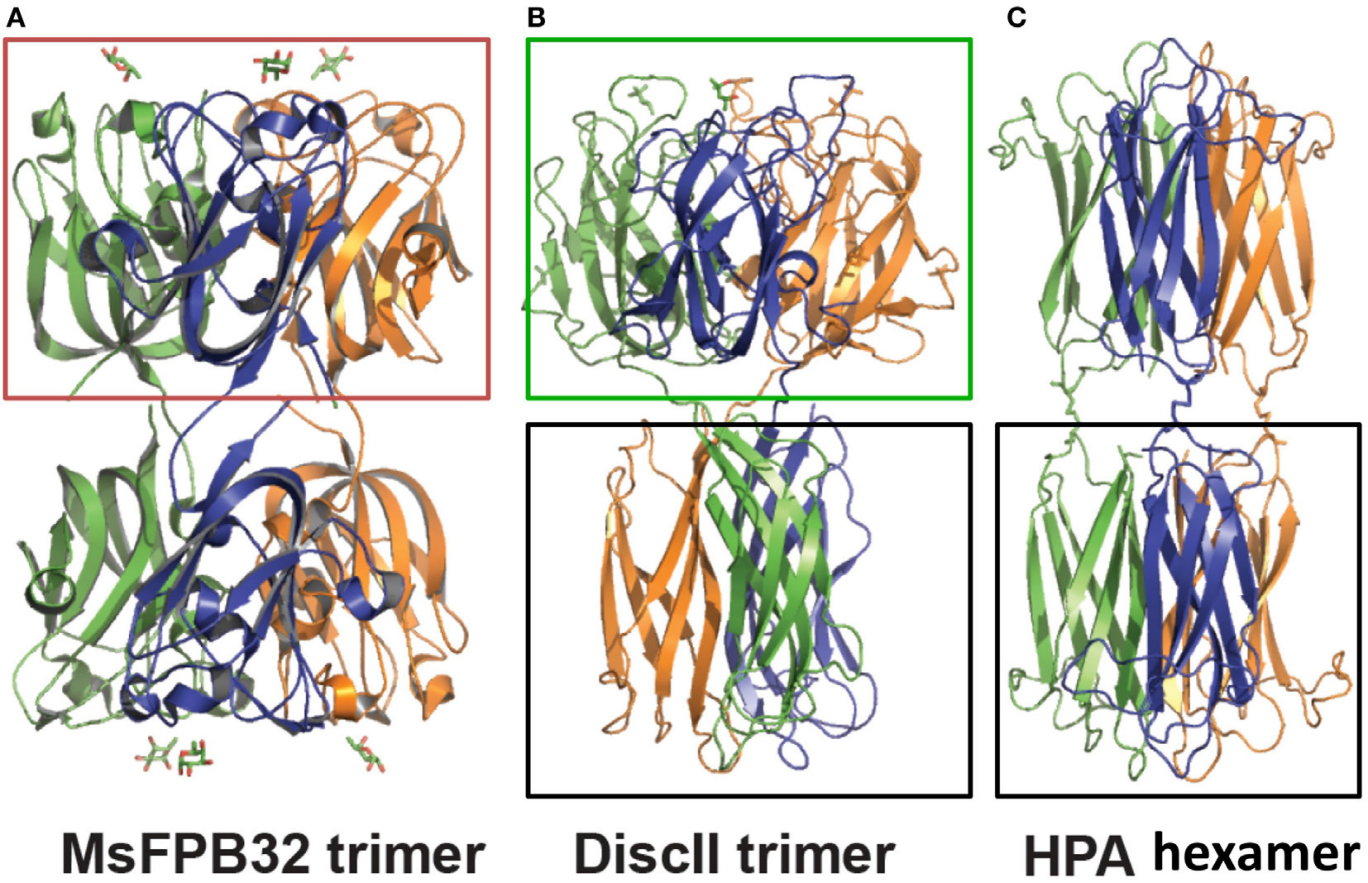
Quaternary structure of tandemly arrayed F-type lectin domains (FTLDs) in MsaFPB32, discoidin II (DiscII), and *Helix pomatia* agglutinin (HPA): Side-by-side comparison between **(A)** MsaFBP32 trimer, **(B)** DiscII trimer, and **(C)** HPA hexamer. FTLD and discoidin domains are boxed on top (brown box in MsaFPB32 and green box in DiscII); H-type lectin domains are boxed in the bottom (black box for DiscII and HPA) [adapted from Ref. (14, 47)].

As described above, MsaFBP32 consists of two tandemly arrayed FTLDs, and in the native oligomer three MsaFBP32 subunits are arranged in a “tail-to-tail” manner (14) (Figure 6A). The resulting MsaFBP32 trimer of approximately 81 Å long and 60 Å wide displays two opposing globular structures, one with the three N-FTLDs and the other with the three C-FTLDs, connected by the linker peptides (14). At the opposite ends of the cylindrical trimer, the 3-CRD binding surfaces resemble the typical “bouquet” displays observed in collectins and can potentially cross-link different humoral or cell surface glycans. Although the N- and C-FTLDs are structurally similar, important differences between their binding sites suggest that the N-FTLD recognizes fucosylated oligosaccharides of higher complexity, with a relatively higher avidity than the C-FTLD (14).

### Other Proteins That Display the FTL Fold

Although the novel FTL fold is distinctive of FTLs in viruses, prokaryotes, and eukaryotes, a structure-based search [DALI database (29)] identified three proteins with no significant sequence similarity to FTLs (2–14% sequence identity with AAA), but shared the jellyroll FTL fold with AAA (13). These sequences correspond to the C1 and C2 repeats of human blood coagulation factor V (37) (FVa-C1 and -C2), the C-terminal domain of a bacterial sialidase (CSIase) (38), and the NH2-terminal domain of a fungal galactose oxidase (NGOase) (39, 40). In addition, other proteins sharing the FTL fold, but with even lower sequence similarities were identified: the human APC10/DOC1 ubiquitin ligase (PDB 1XNA) (41), the N-terminal domain of the XRCC1 single-strand DNA repair complex (PDB 1JHJ) (42), and a yeast allantoicase (PDB 1SG3) (43). An alignment of the CSIase, NGOase, FVa-Ca, and AAA sequences showed that residues equivalent to the Asp^64^, Pro^106^, and Arg^131^ are strictly conserved. In the four structures, the core and the bottom of the β-barrel are very similar, with the loops at the top varying in length and conformation. In CSIase and NGOase, two members (His and Arg) of the triad of basic residues that interact with the axial hydroxyl of fucose in AAA are conserved. CSIase, the galactose-binding domain of the bacterial sialidase, has been shown to bind carbohydrate (38). Furthermore, in NGOase also two residues (His^40^ and Arg^73^) homologous to those involved in carbohydrate recognition by AAA (His^52^ and Arg^79^) and in CSIase (His^539^ and Arg^572^) are conserved, suggesting that it may bind carbohydrate. In FVa-C2, all residues of the basic triad related to carbohydrate binding are absent, making this pocket the most hydrophobic and the deepest. Interestingly, FVa-C2 has affinity for phospholipids instead of carbohydrates (37). Thus, these observations provide potentially useful clues either about the evolutionary history of FTLs as emerging from carbohydrate-binding domains in glycoenzymes or suggest that the recognition properties of the FTLs have been drastically modified or coopted to bind membrane phospholipids (37–40).

## TAXONOMIC DISTRIBUTION AND EVOLUTIONARY ASPECTS OF THE FTLD

The initial recognition of FTLs as a novel lectin family resulted from the identification and characterization of the FTLD sequence motif in taxa ranging from prokaryotes to amphibians (11, 12) and the identification of the F-type structural fold (13). These studies identified the FTLD sequence motif in lophotrochozoan (mollusks and planaria) and ecdysozoan protostomes (horseshoe crabs and insects), deuterostome invertebrates (sea urchin), elasmobranchs (skate), lobe- and ray-finned teleost fish, and amphibians (*Xenopus* spp. and salamander) (11, 12). However, intriguing observations in these earlier studies such as the discontinuous taxonomic distribution, and diversified domain architecture of the FTL family members, frequently in combination with other structurally distinct domains, pointed to a functionally plastic FTLD, which had been specifically tailored in each lineage, subjected to lateral transfer, and that either enhanced or lost its fitness value in some taxa (11, 12). The absence of the FTL sequence motif in archaea, protozoa, urochordates, and higher vertebrates suggested that it may have been selectively lost even in relatively closely related lineages (11, 12, 30).

The advent of innovative sequencing technologies during the last decade has enabled comprehensive genomic and transcriptomic studies on a large variety of organisms and significantly expanded our knowledge about the taxonomic distribution of the FTLD from viruses to prokaryotes and eukaryotes. In this regard, a rigorous and exhaustive computational study on publically available databases by Bishnoi et al. has recently provided significant insight and greatly expanded the range of taxa in which the FTLD is found (30). Using a three-pronged database mining approach, Bishnoi et al. identified FTLDs for the first time in viruses, fungi, reptiles, birds, and prototherian mammals (30). Furthermore, their study confirmed the diversity observed in mollusks (24–26) and revealed a substantial expansion in both FTLD occurrence and domain organization diversity in hemichordates and cephalochordates. Consistently with the aforementioned earlier reports (11, 12, 30), however, the study revealed that FTLDs appear to be absent in archaea, protozoans, urochordates, and eutherian mammals. Furthermore, no FTLDs were identified in higher plants (30).

**Figure 7:**
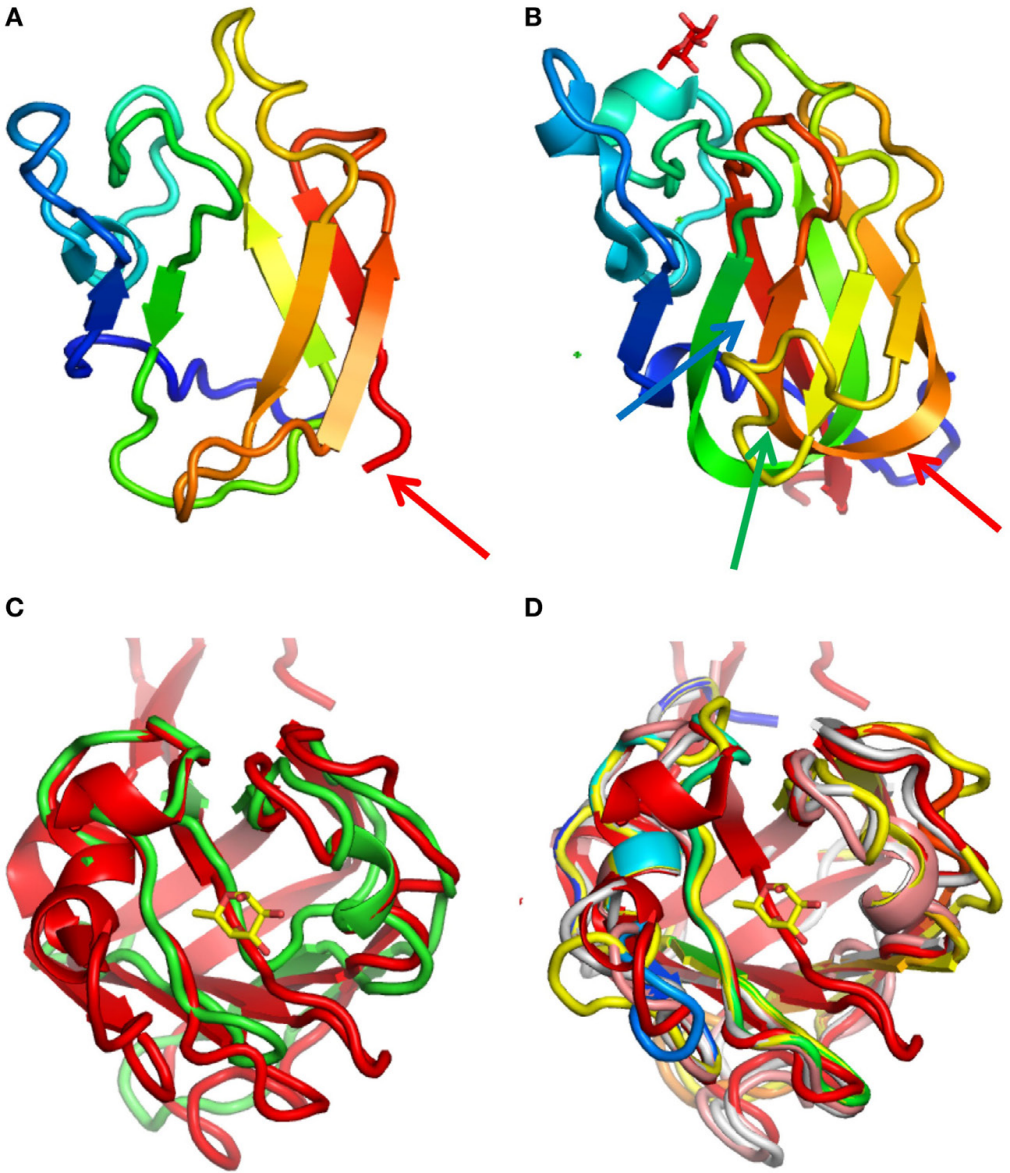
Structural models of viral F-type lectin domains (FTLDs): **(A,B)** lateral views of the model of a viral FTLD (*Ostreococcus lucimarinus* OlV1) **(A)** and the structure of *Anguilla anguilla* agglutinin (AAA) **(B)**. The traces are colored from the blue N-terminal, and the red arrows show the C-terminal of the viral protein and the position of the equivalent residue in the AAA structure. The green (AAA residues 126–136) and blue (AAA residues 145–155) arrows point to two strands of the AAA fold that are missing in the viral protein, of which the former strand (indicated by the green arrow) is structurally very important. **(C,D)** Superposition of the model of the viral FTLD from *Emiliania huxleyi* virus 203 (green) with the AAA structure (red). **(C)** *E. huxleyi* virus 203 contains a strand (indicated in green) that forms the floor of the cavity of the binding site and that overlaps with the AAA structure (red). **(D)** This strand is missing in all other viral (from *Ostreococcus* spp.) FTLDs.

From over 400 FTLD sequence clusters (at 80% sequence identity) tentatively identified in available databases by Bishnoy et al. (30), six FTLD sequence clusters from dsDNA viruses isolated from unicellular algae were confirmed, five from the chlorophyceans *Ostreococcus* sp., *O. tauri*, and *O. lucimarinus* [*O*. sp. virus OsV5, *O. tauri* virus 1 (two distinct sequences), *O. lucimarinus* OlV1, and *O. lucimarinus* OlV6], and one from the coccolithophore *Emiliania huxleyi* (*E. huxleyi* virus 203), which are microalgal species abundant in photosynthetic phytoplankton. Except for a viral FTLD joined by a PTX domain found in the *E. huxleyi* virus 203, all other viral FTLD sequences are single. It is noteworthy that although *E. huxleyi* and *Ostreococcus* spp. also display FTLDs, some with high similarity to the viral FTLDs, the microalgal host’s FTLDs are associated with other distinct non-FTLD domains (30). The structural models of the viral FTLDs threaded on the AAA structure (13) revealed interesting features (Figures 7A–D). First, all viral FTLDs display the triad of basic residues (His, Arg, and Arg) that interact with the hydroxyl on C4 of L-Fuc, with the exception of *E. huxleyi* virus 203 that has only Arg-Arg. Furthermore, they all display phenylalanine instead of the disulfide bond between contiguous cysteines (Cys^82^ and Cys^83^ in AAA) that in AAA interacts with the bond between ring atoms C1 and C2 of α-Fuc. Second, two strands of the AAA fold (AAA residues 126–136 and residues 145–155), of which the former strand (indicated by the green arrow in Figure 7B) is structurally very important, are missing in the viral proteins (Figure 7A). It is not clear whether the FTLD structure without this strand would be stable, and it is possible that in the expressed protein the sequence corresponding to this strand might be inserted by a splicing event that was not detected in the DNA sequencing. Additionally, in the model for *E. huxleyi* virus 203 FTLD, a strand that forms the floor of the cavity of the binding site tightly overlaps with the equivalent strand in the AAA structure (Figure 7C). This strand, which in AAA connects the last two strands of the β-barrel, is also missing in all other viral FTLDs (Figure 7D). Interestingly, the viral FTLDs cluster with those from several other microalgal species (*Volvox* sp. and *Chlorella* sp.) and with several oyster (*Crassostrea* spp.) and mussel (*Mytilus* sp.) species (30). The fact that oysters and mussels are filter feeders that actively uptake phytoplankton together with any associated viruses supports the possibility of horizontal transfer between bivalves, microalgae, and their viruses. Diatoms, cryptomonads, brown algae, green algae, and fungi (*Phytophthora* spp.) also possess singly or tandemly arrayed FTLDs, mostly associated with other structurally and functionally distinct domains, but no FTLDs were identified in higher plants (30).

**Figure 8:**
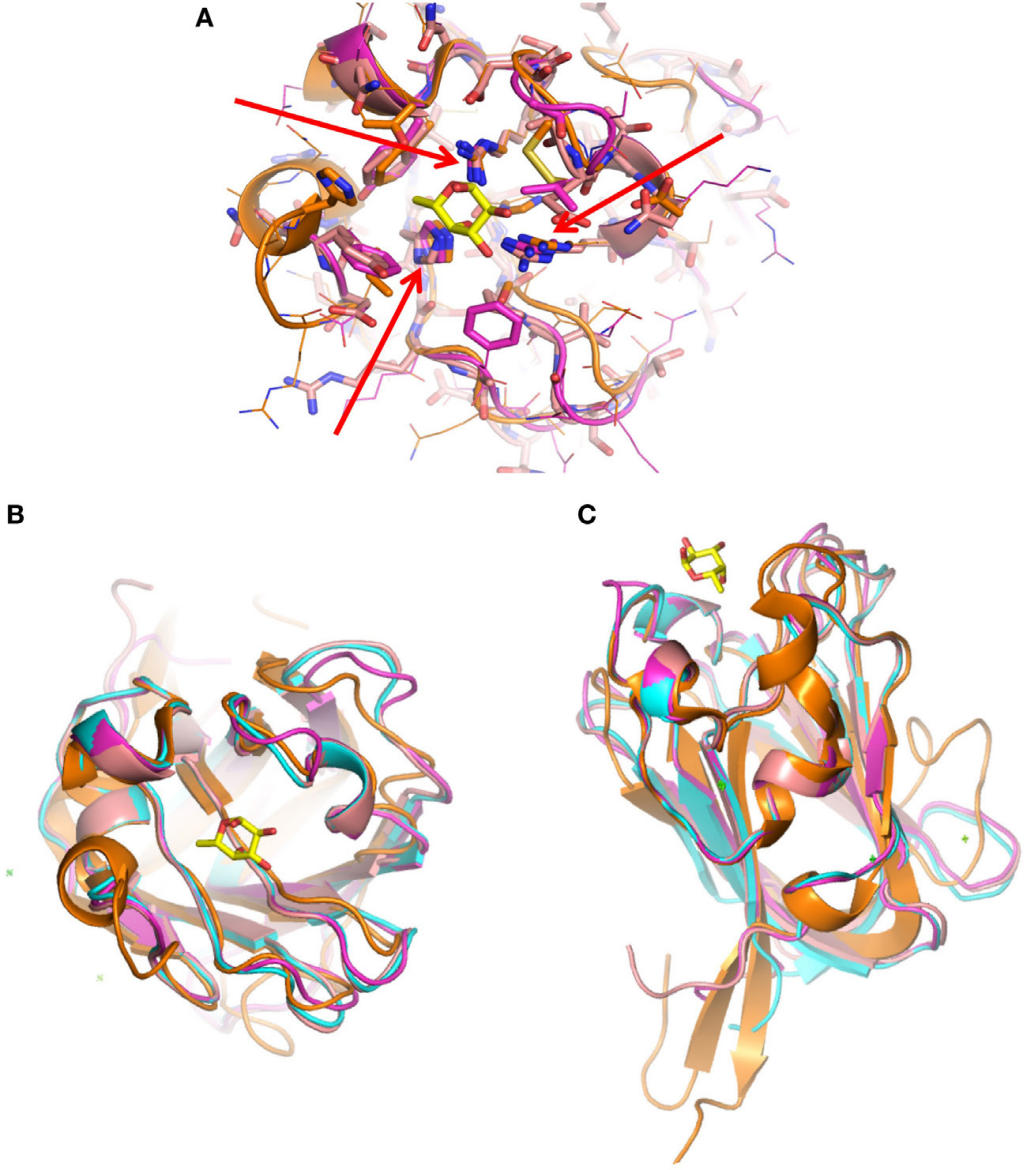
Structural models of F-type lectin domains (FTLDs) from *Streptococcus* spp. **(A)** Superposition of structural models *Streptococcus pneumoniae* (GA41301 1.2 and 1.1) and *Streptococcus mitis* FTLDs with the structure of *Anguilla anguilla* agglutinin (AAA): the red arrows show the conserved triad of basic residues (His-Arg-Arg) in the three streptococcal FTLDs, which in the AAA structure were shown to interact with the hydroxyl on C4 of l-fuc (orange: His^52^, Arg^79^, and Arg^86^) to provide α-l-fucose (α-Fuc) specificity. Bound α-Fuc is shown as a stick model in yellow. The other residues in the primary and extended carbohydrate-binding sites of AAA are not conserved among the three streptococcal FTLDs and could reflect recognition of different fucose-containing oligosaccharides. However, the structure of *S. pneumoniae* FTL determined in complex with the blood group H-trisaccharide shows almost no additional interactions besides those with the α-Fuc. **(B,C)** Variability of the CDRs in FTLDs from *Streptococcus* spp.: top and lateral views of the FTLD fucose-binding site: the superposition of the models shows that most of the variability resides in the loops (CDRs) that encircle the binding cleft. AAA (orange), *S. pneumoniae* 1.2 (light blue), *S. pneumoniae* 1.1 (violet), and *S. mitis* (light purple/orchid). Bound α-Fuc is shown as a stick model in yellow.

**Figure 9:**
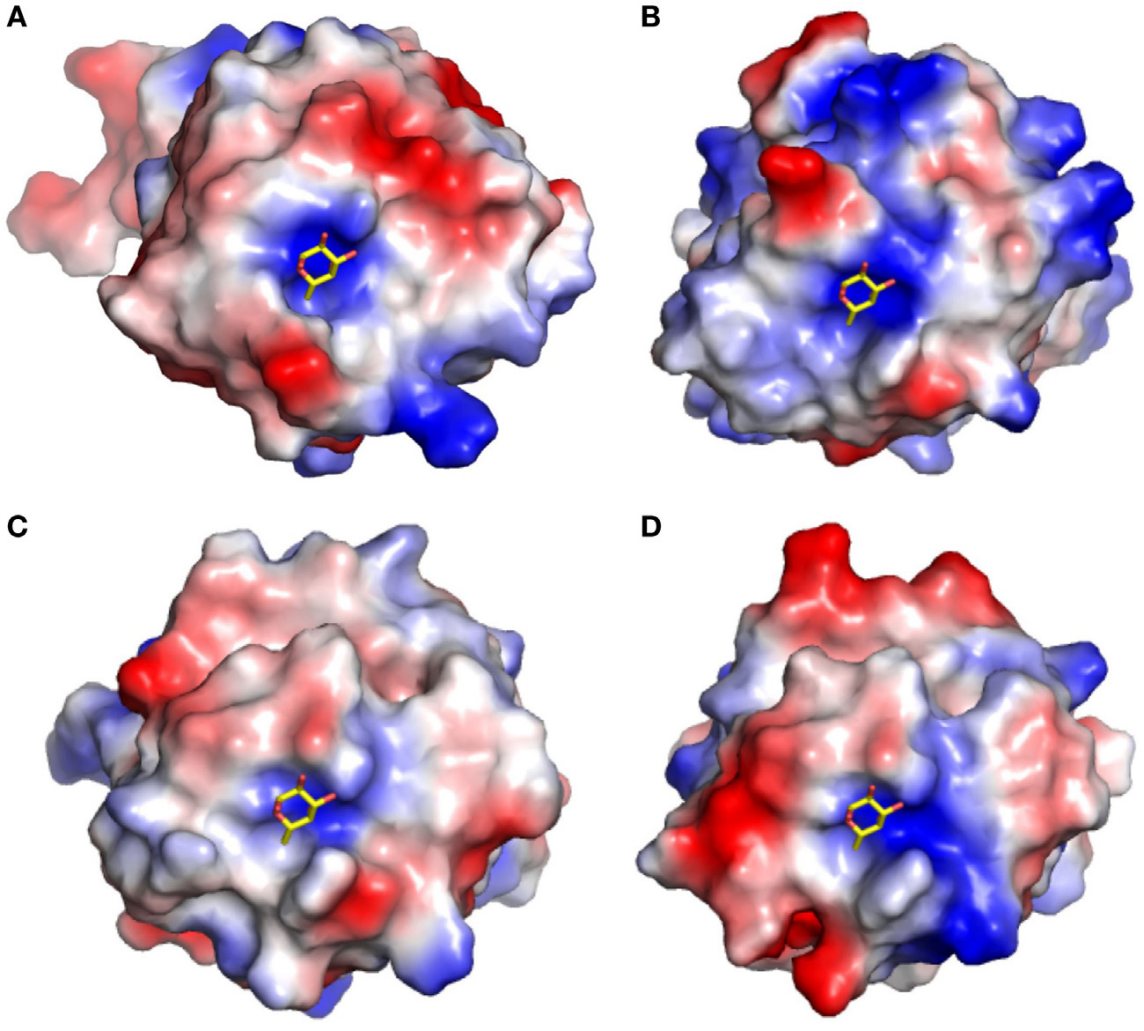
Surface electrostatic potential surfaces of F-type lectin domains (FTLDs) from *Streptococcus* spp.: **(A)** *Anguilla anguilla* agglutinin (AAA) structure, **(B)** *Streptococcus mitis*, **(C)** *Streptococcus pneumoniae* 1.1, and **(D)** *S. pneumoniae* 1.2. The electrostatic surfaces show that the positively charged binding cleft for the α-l-fucose ligand is highly conserved in all three streptococcal FTLDs as compared to AAA, but the charge characteristics of the surrounding residues in the CDRs are highly variable.

In prokaryotes, FTLDs were initially identified in a hypothetical protein (GenBankTM accession number ZP_00065873) from *M. degradans*, a Gram-negative bacterium with broad polysaccharide substrate degrading capability (33) and a gene (GenBank™ accession number AE007504) that is part of the l-Fuc catabolic regulon (44) in the Gram-positive *S. pneumoniae*. In the latter, three tandemly arrayed FTLDs were identified. Later studies characterized the *Streptococcus* spp. FTLDs as carbohydrate-binding domains of cholesterol-dependent cytolysins (CDCs), a large family of pore-forming and platelet-aggregating toxins (20–23). Comparison of structural models of *S. pneumoniae* (GA41301 1.2 and 1.1) and *S. mitis* FTLDs with the structure of AAA revealed the conserved triad of basic residues (His-Arg-Arg) that in the AAA structure (His^52^, Arg^79^, and Arg^86^) were shown to interact with the hydroxyl on C4 to provide α-Fuc specificity. The other residues in the primary and extended carbohydrate-binding sites of AAA are not conserved among the three streptococcal FTLDs and could reflect recognition of different fucose-containing oligosaccharides (Figure 8A). However, the structure of *S. pneumoniae* FTL determined in complex with the blood group H-trisaccharide shows almost no additional interactions besides those with the α-Fuc. Furthermore, the superposition of the models shows that most of the variability resides in the loops (CDRs) that encircle the binding cleft (Figures 8B,C). This is supported by the electrostatic potential of the FTLD surfaces that show that the positively charged binding cleft for the α-Fuc ligand is highly conserved in all three streptococcal FTLDs as compared to AAA, but the charge characteristics of the surrounding residues in the CDRs are highly variable (Figures 9A–D).

Bishnoi et al. (30) recently identified FTLDs in several additional prokaryotic taxa (i.e., Actinobacteria, Bacteroidetes, Planctomycetes, Firmicutes, Proteobacteria, Cyanobacteria, Verrucomicrobia, and others) both as single or replicate FTLDs, in most cases associated with distinct sequences that included domains from other carbohydrate-binding proteins, as well as glycoenzymes, lipases, methyltransferases, and other enzymes (30). This observation is suggestive of environmental adaptations of prokaryotes for the catalytic modification of glycosylated substrates. Furthermore, the intermittent distribution of FTLDs in prokaryotic taxa suggests either their acquisition from metazoans through horizontal transfer, or less likely, that many prokaryote lineages or taxa suffered a secondary loss of the FTLD (11, 12).

The discoidins I and II (DiscI and DiscII) from the slime mold (*Dictyostelium discoideum*) are trimers of protein subunits that carry two distinct lectin domains: an N-terminal “discoidin” domain that displays the FTL fold (Figures 6A,B) and a C-terminal lectin domain structurally similar to the snail *Helix pomatia* lectin (HPA) H-type domains (Figures 6B,C) (45–47). The oligomeric organization of discoidins and *H. pomatia* lectin strongly resembles the trimeric structure of the MsaFBP32 (47) (Figure 6A). Although, discoidins are reported to bind GalNAc, as expected from the presence of the H-type domain, their potential binding to fucosylated ligands has been recently analyzed in a glycan array (47).

Among the invertebrate taxa, FTLDs have also been identified in those species for which abundant genomic or transcriptomic information is available, either due to their long-standing evolutionary, ecological, or commercial interest, their use as effective model systems, or their biomedical relevance. In cnidarians such as the freshwater hydra, *Hydra vulgaris*, the FTLD is associated with a CTLD, while in the marine anemone *Nematostella vectensis*, it forms part of a complex protein that carries CCP, EGF-like, and other distinct domains (30). FTLDs were also identified in worms, including the nematode *Caenorhabditis elegans* and the annelid *Capitella teleta* (30). In arthropods, FTLDs have been identified either associated with multiple non-FTL domains or as standalone domains. For example, among chelicerates, FTLDs are present in the tachylectin from the horseshoe crab *Tachypleus tridentatus* (11, 12, 16, 30), and in the tick *Ixodes scapularis* (30), while in crustaceans FTLs were found in the prawn *Macrobrachium rosenbergii* (30). As discussed above, FTLDs were initially identified in insects as the *furrowed* gene from the fruit fly *Drosophila*, and in the mosquito, *Anopheles gambiae* (GenBank accession # AAAB01008846 and AAAB01008811) (11, 12). In mollusks, FTLDs have been well characterized in the highly diversified oyster bindins discussed above (24–26), as well as other oyster species, mussels, and clams (30, 48–52). Similarly, an extraordinary expansion in prevalence and organizational complexity of the FTLD were noted among protochordates, specifically in the hemichordates (acorn worms) and cephalochordates (over 70 different FTLD sequence clusters in the amphioxus *B. floridae*) (30), but surprisingly, they appear to be absent in urochordates (i.e., ascidians and salps) (11, 12, 30).

The initial studies revealed that the substantially diverse FTLD organizational topologies in cold-blooded vertebrates, such as fish and amphibians, appear to be in some cases lineage-related (11, 12). As the F-type fold displays joined N- and C-terminals, this structural feature promotes the assembly of multiple CRD topologies that are consistent with phylogenetic clustering. In this regard, the binary FTLs have diversified through lineage-dependent gene duplications that are unique to teleosts and amphibians (11, 12). For example, most teleost FTLs contain either two or four tandemly arrayed FTLDs, whereas in *Xenopus* spp. FTLs are organized from single FTLDs to combinations of two, three, or four FTLDs and as chimeric proteins containing five tandemly arrayed FTLDs adjacent to a PXN domain (11, 12). The study by Bishnoi et al. (30) identified additional CTLD and the clotting factor FA58C domain associated with FTLDs in teleost fish, including the coelacanth *Latimeria* sp., and for the first time identified FTLDs in reptiles, birds, and mammals, in the latter associated with PXN domains (30). Interestingly, FTLDs were only identified in prototherian mammals, including the monotremes, such as platypus, and didelphid marsupials, such as the opossum (30), but appear to be absent in eutherian (placental) mammals (11, 12, 30).

## FUNCTIONAL ASPECTS

In spite of the broad range of taxonomic distribution of FTLDs, their functional properties have only been experimentally demonstrated in a limited number of examples. In most cases, their biological roles have been rather inferred from their gene expression levels and cell- or tissue-specific localization upon experimental immune challenge or environmental stressors, together with their structural features, biochemical properties, including their binding selectivity for endogenous and microbial glycosylated ligands. In those few examples in which FTLDs have been studied in genetically tractable model systems, such as the streptococcal lectinolysins, their roles have been rigorously established not only by genetic approaches but also by significant contributions of the rigorous analysis of their structures (20–23). In *Drosophila*, however, although the role of the *furrowed* gene in cell adhesion was clearly established, the specific function of the FTLD in this process remains to be elucidated (11, 12, 31). In the slime mold *D. discoideum*, a widely recognized genetically tractable model system for developmental and cell biology studies, the role(s) of DiscI and DiscII remains to be rigorously established (47). Although initially both discoidins were reported as secreted lectins involved in cell–substratum adhesion and spore coat formation (53, 54), later studies questioned these results as no evidence of their secretion could be found. A recent study, however, concluded that DiscI is implicated in cell–substratum adhesion and plays a role in streaming (55) although the mechanistic aspects have not been elucidated yet.

Initially identified and characterized in teleost fish, the multivalent FTLD display and their distinct carbohydrate specificity revealed the clear potential of oligomeric FTLs for binding to microbial surface glycans (13, 14). For example, both the trimeric arrangement of the AAA FTLDs and the opposite orientation of the distinct N- and C-terminal binding surfaces of the trimeric MsaFBP32 strongly suggest that in circulation these lectins can cross-link fucosylated glycoconjugates displayed on different cells (13, 14). Modeling of the MsaFBP32 recognition of fucosylated oligosaccharides from prokaryotes and eukaryotes supports the observation that FTLs with binary tandem CRDs can function as opsonins (14). Opsonization of potential pathogens would take place by FTL-mediated cross-linking exposed carbohydrate moieties on microbial pathogens with surface glycans on the host’s phagocytic cells (14). By recognizing Le^a^-containing glycans on the phagocytic cell surface *via* the N-CRD, MsaFBP32 would cross-link the infectious agent *via* the C-CRD, which recognizes glycans α-linked l-Fuc, 2-acetoamido l-Fuc, 3-deoxy-l-fucose (colitose) or l-Rha (6-deoxy-l-mannose, present in *Escherichia coli* glycans) as non-reducing terminal residues on the microbial surface (14). The tissue expression of fish FTLs that primarily takes place not only in liver (11, 12, 56–60), the typical source of acute phase reactants, but also in gills (15, 56–58) and intestine (56–58), which are organs continuously exposed to infectious challenge, is highly suggestive of their role(s) in innate immune defense. The opsonic properties of FTLs were experimentally demonstrated with the binary tandem FTLs from sea bass (DlFBL; *Dicentrarchus labrax*) and gilt head bream (SauFBL; *Sparus aurata*) (58, 60). Pre-exposure of *E. coli* to DlFBL or SauFBL significantly increases their uptake by peritoneal macrophages as compared to the unexposed bacteria (58, 60) supporting the concept that F-lectins with multivalent FTLs such as AAA, DlFBL, SauFBL, and MsaFBP32 can function as opsonins that promote phagocytosis of microbial pathogens. By transfecting the EPC cell line with an FTL (RbFTL-3) that is highly expressed in the intestine of rock bream (*Oplegnathus fasciatus*), followed by viral (viral hemorrhagic septicemia virus) challenge, Cho et al. (61) recently showed that RbFTL-3 controls viral budding and increases the viability of VHSV infected cells, suggesting that the lectin limits hemorrhage in fish tissues.

Upregulation of FTL expression by immune challenge as it would be expected by analogy to liver expression of acute phase reactants in innate immune responses, however, has not been the general rule for the species examined. For MsaFBP32, an inflammatory challenge only increased the liver transcript levels in about three-fold over the relatively high basal expression levels (11, 12), whereas for DlFBL protein levels were modestly enhanced by *Vibrio alginolyticus* infectious challenge (58). In the Japanese sea perch (*Lateolabrax japonicus*), the FTL JspFL was only upregulated in spleen, while it was also constitutively expressed in liver and gills (62). In contrast, LPS challenge significantly upregulated expression and increased secretion of FTLs in liver and gill tissue from *A. japonica* (15).

As FTLs have not only been identified in the eukaryotic hosts but also in viral, prokaryotic, and multicellular pathogens and parasites, the intriguing possibility that FTLs may play key roles in microbial virulence has only been examined in detail in bacterial lectinolysins (17–23, 63). It is widely recognized that opportunistic bacteria recognize and attach to host cell glycans *via* carbohydrate-binding domains in their surface proteins (64, 65). However, Gram-positive bacteria (18) such as *Streptococcus* spp. (*S. pneumoniae, S. mitis*, and *S. intermedius*) and *Garnderella vaginalis*, among others, produce CDCs (lectinolysin, pneumolysin, intermedilysin, and vaginolysin) that bind to and disrupt the host cell membrane (17–23). The *S. mitis* lectinolysin, also described as a platelet aggregation factor (17), carries an FTLD that recognizes the host’s fucosylated moieties to significantly enhance their virulent pore-forming properties in at least one order of magnitude. Upon binding to the host surface glycans, monomeric CDCs spontaneously self-assemble to form large β barrel pores that lead to cell lysis (63). The FTLD of the CDC specifically recognizes difucosylated glycans [Lewis y (Le^y^) and Lewis b (Le^b^) moieties], and it has been controversial whether the fucose-binding site remains masked in the CDC monomer and is only exposed following contact with the cell surface (21), or if it is fully accessible to the environment and ready for interaction with host cell glycoreceptors (22).

In contrast with the innate immune host defense and the bacterial virulence functions of the FTLDs described above, the sperm “bindins” from the Pacific oyster (*C. gigas*), discussed in a previous section, are highly polymorphic proteins stored in the acrosomal rings of sperm cells that bind to the surface of the egg perivitelline envelope during fertilization (24). By mechanisms of positive selection, recombination, and alternative splicing, a single copy bindin gene can produce transcripts that are highly diversified both in sequence and domain organization within and among individuals in this oyster species. Interestingly, each individual male oyster will translate only one or two polymorphic bindins carrying between one and five tandemly arrayed F-lectin domains are translated in Ref. (25). The unusual high intraspecific diversity of the oyster bindin F-lectins has been proposed to represent coevolution of sperm gamete recognition mechanisms to “catch-up” with the high diversification of egg receptors aimed at avoiding polyspermia (26). It should be noted, however, that FTLs have also been reported as defense molecules in oysters (48–52) and several other invertebrate species. Among these, an FTL (PmF-lectin) from pearl oyster (*Pinctada martensii*) is highly expressed in hemocytes and gill and significantly upregulated (13-fold) by infectious challenge (*V. alginolyticus*), suggesting that PmF-lectin is involved in the innate immune response (48). The highly diversified FTL repertoire identified in the common periwinkle (*Littorina littorea*) has been hypothesized as an immune defense system (52), whereas in the blunt-gaper clam *Mya truncata*, FTLs have been identified in both the shell matrix and mantle tissue proteins, suggesting that during the shell biomineralization process, immune defense functions may be carried out by proteins secreted by the mantle, which are later incorporated into the shell matrix (51).

## CONCLUSION

**Figure 10:**
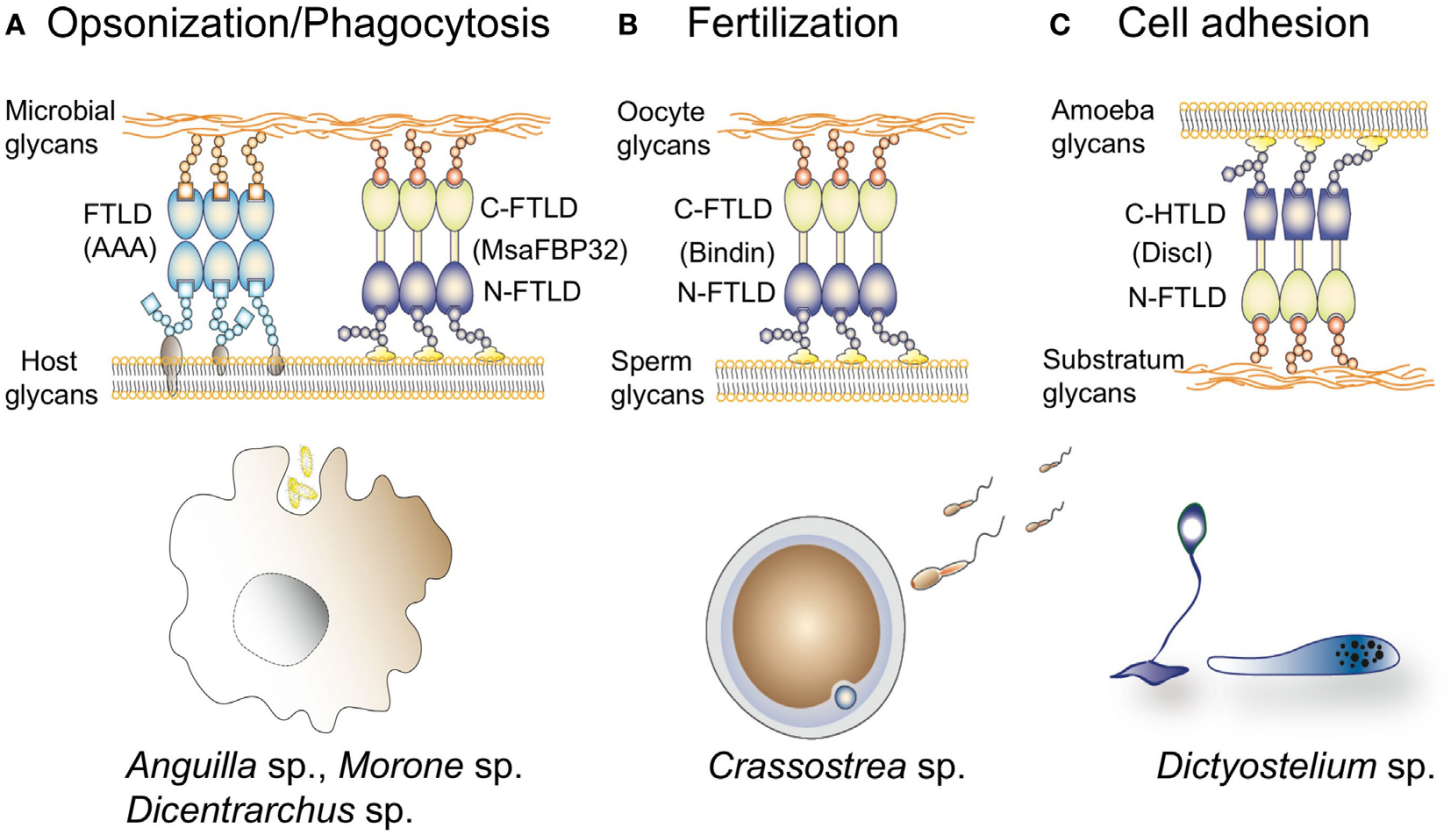
Schematic illustration of hypothesized self- and non-self-recognition by F-type lectins (FTLs) in immunity, fertilization, and cell adhesion: The cartoon illustrates the potential binding activities of FTL domains (FTLDs) identified in fish [*Anguilla anguilla* agglutinin (AAA) and MsaFBP32], oyster (bindins), and slime mold (DiscII) discussed in the text: **(A)** Both AAA and MsaFBP32 oligomers are multivalent and can cross-link glycans on the surface of potential microbial pathogens to the surface of the macrophages, leading to opsonization, phagocytosis, and intracellular killing of the infectious agent. **(B)** The highly diversified oyster (*Crassostrea gigas*) bindins, carrying up to five FTLDs, may selectively cross-link sperm or acrosomal glycans to the egg perivitelline envelope enabling only fertilization by sperm that matches the egg glycans and prevent polyspermia. **(C)** Discoidins secreted by the slime mold (*Dictyostelium discoideum*) ameba may cross-link surface glycans to substratum components, enabling cell–substratum adhesion and streaming.

The structural and functional analyses of the FTLD, together with its distribution in extant viral, prokaryotic, and eukaryotic species reveal an intriguing evolutionary history of this lectin domain with key adaptations to a diverse array of functions carried out by the FTLD itself, either as single units or as tandemly arrayed domains. This functional diversity is further expanded for FTLDs associated with structurally and functionally distinct associated domains, either belonging to other lectin families (CTLs and PXNs), enzymes, or other proteins. Thus, FTLs are essentially pleiotropic and can orchestrate a vast array of functions based on “self” and “non-self”-recognition that encompass not only innate immunity but also fertilization, cell adhesion, and microbial virulence, among others yet to be unraveled (Figure 10). In recent years, a substantial body of evidence has supported the proposal that along their evolution, selected FTLs were co-opted to carry out different functions that may not rely on active carbohydrate-binding sites, and therefore, this property, which is inherent to their definition as lectins, may have been lost in the process. The paucity in the taxonomic distribution of viral and prokaryotic FTLDs suggests the eukaryotic origin of the domain, followed by extensive duplication and mutation, lateral transfer, and secondary loss or cooption (11, 12). This is supported by the observation that a phylogenetic analysis revealed that although in general the clustering of FTLDs is consistent with the taxonomical categories, bacterial FTLDs are interspersed with several eukaryotic FTLDs (30). Furthermore, the viral FTLDs cluster with those from several other microalgal species (*Volvox* sp. and *Chlorella* sp.) and with several oyster (*Crassostrea* spp.) and mussel (*Mytilus* sp.) species (30). In this regard, it is important to note that oysters and mussels are filter-feeder bivalves that actively uptake microalgae (together with their associated viruses) from the suspended phytoplankton, thereby providing clues about the origins and potential lateral transfer of the viral, microalgal, and mollusk FTLDs.

On the other hand, despite the FTL diversity evident in amphibians, reptiles, birds, and prototherian mammals, no *bona fide* FTL homologs are detectable in genomes of eutherian mammals (11, 12, 30). Therefore, above the level of the prototherian mammals this lectin family may have been lost as such, either by becoming truly extinct or by being co-opted into other functions as proposed for the C-1 and C-2 domains of the clotting factors V and VIII (11–13). While lacking carbohydrate binding capacity due to the loss of the triad of basic residues that interact with the axial hydroxyl of the sugar ligand, the aforementioned C-1 and C-2 domains still display the F-type fold and are highly prevalent not only in taxa ranging from fish to birds but also widespread in eutherian mammals (11–13). It is possible that the loss of fucose recognition activity has been driven by the need to avoid self-reactivity to fucosylated moieties exposed on the cell surface, such as the blood group H and Lewis oligosaccharides that arose along the eutherian mammal lineages.

With regards to their roles in immune recognition, as described above FTLs can display in a single polypeptide monomer single or tandemly arrayed CRDs of similar but distinct specificity. Therefore, cross-linking of “self” and “non-self” carbohydrate moieties can be easily rationalized by: (a) the different specificity of their binding sites, (b) the distinct architecture of the presentation and multivalency of the carbohydrate ligands on the microbial cell surface or the host, and (c) the biophysical properties of the microenvironment where the interactions occur (11–14). Bishnoi et al. identified a substantial expansion in both FTLD occurrence and domain organization diversity in the mollusks, hemichordates, and cephalochordates that was attributed to enhanced emphasis on innate immunity in these taxa (30). Consistently with earlier studies (11, 12), however, the study revealed that FTLDs are absent in urochordates (ascidians and salps) (30). First of all, the FTL diversification observed in mollusks is most likely due at least in part to their expanded functions as gamete recognition molecules in fertilization processes (“bindins,” described above) (24–26). Second, it is well established that urochordates, like hemichordates and cephalochordates, also lack *bona fide* adaptive immune systems such as the variable lymphocyte receptors (VLRs) and immunoglobulin- and B/T cell-mediated immune responses and solely rely on innate immunity for defense against infection. Thus, the increased FTLD diversification in hemichordates and cephalochordates, together with the lack of FTLDs in urochordates could be rather attributed to compensatory effects among multiple lectin families, depending on selective advantage(s) that each can provide to any given taxa as more or less effective pattern recognition receptors in innate immunity. In support of this view, it is noteworthy that the urochordata ascidian *Clavelina picta*, which lacks FTLs, expresses a highly diversified repertoire of fucose-binding CTLs, suggesting that the expansion of the CTL repertoire probably reflects the selective advantage that fucose-binding CTLs provides over FTLs to the ascidian’s innate immune responses (66, 67). In addition to functions carried out by FTLs, such as pathogen recognition, immobilization, and opsonization, CTLs can also initiate complement activation, an ancient enzyme-driven mechanism that can rapidly amplify opsonization and effect direct killing of the potential pathogen *via* the membrane attack complex (66, 67). Therefore, it is possible that these and other functional advantages offered by CTLs led to their expansion as innate immune defense mechanisms in higher mammals, simultaneously with the contraction, cooption, or loss altogether of the FTL family members.

The rapidly expanding genomic databases and their increasing availability for numerous animal species have provided further insight into the structural and functional diversification of lectin repertoires from prokaryotes, invertebrates, protochordates, and vertebrates. In this context, the recent identification of novel lectin families such as the FTLs (11–14), underscores the need of more research in non-mammalian model organisms. This will provide greater insight into the structural, functional, and evolutionary aspects of lectin families that may not be as obvious in the traditional mammalian model systems. In this regard, the structural analysis of multiple FTL isoforms in eels and oysters (15, 24–26) has revealed substantial diversity in oligosaccharide recognition and has provided conceptually transformative insight into the processes through which lectins can generate an extraordinary structural and, most likely, functional diversity for self/non-self-recognition that resembles those mechanisms operative in adaptive immunity of higher vertebrates. The current exponential increase in the genome, transcriptome, and proteome information on additional non-mammalian model organisms, coupled with structural studies and innovative forward and reverse genetic approaches for functional analyses has the potential to uncover novel structural, functional, and evolutionary features in various lectin families, from viruses and prokaryotes to mammals. Furthermore, homology modeling of novel FTLs on related crystal structures will contribute to rapidly expand our knowledge about their interactions with potential glycosylated ligands (68). Due to its substantial advantages over mammalian models, namely external fertilization, transparent embryos, a continuously expanding collection of mutations and a rapidly growing toolbox for manipulation of gene expression, the zebrafish may constitute an ideal model for the elucidation of the biological roles of FTLs in innate immunity of vertebrates. Finally, given the prevalence of fucosylated moieties on the surface of neoplastic cells, it is possible that FTLs may become useful reagents for both diagnostics and therapeutic applications in cancer (69, 70).

## AUTHOR CONTRIBUTIONS

GV designed, drafted, and edited the final manuscript; LA and MB developed and analyzed structural models, evaluated and edited the draft manuscript; and MC, CF, and KS evaluated and edited the draft manuscript.

## Conflict of Interest Statement

The authors declare that the research was conducted in the absence of any commercial or financial relationships that could be construed as a potential conflict of interest.
